# Juvenile Vogt-Koyanagi-Harada Disease in Which Good Visual Prognosis Was Derived from Swift and Definitive Diagnosis

**DOI:** 10.1155/2016/7936729

**Published:** 2016-03-27

**Authors:** Atsushi Yoshida, Satoko Tominaga, Hidetoshi Kawashima

**Affiliations:** Department of Ophthalmology, Jichi Medical University, 3311-1 Yakushiji, Shimotsuke, Tochigi 329-0498, Japan

## Abstract

We report an 8-year-old girl who manifested Vogt-Koyanagi-Harada (VKH) disease. At the first visit, conjunctival hyperemia, inflammation in the anterior chamber, serous retinal detachment, and papillitis were observed in both eyes. Fluorescein angiography (FA) revealed bilateral subretinal fluid and papillitis. Ocular computed tomography (OCT) showed subretinal fluid and choroidal hypertrophy underneath macula in both eyes. Cerebrospinal fluid examination indicated aseptic meningitis. Systemic data did not suggest the other systemic diseases. Therefore, she was diagnosed with incomplete VKH disease. After corticosteroid pulse therapy, oral prednisolone was administered for seven months. Eighteen days after the induction of the treatments, inflammation in the anterior chamber and serous retinal detachment of both eyes disappeared completely. For seven months after the induction of the treatments, she had no relapses of any symptoms. Cerebrospinal fluid examination and FA for children are difficult to conduct, since it is difficult to get informed consent of these examinations from their parents. However, those thorough examinations enable us to make a swift and definitive diagnosis of VKH disease, thus assuring good visual prognosis. We have to bear in mind that juvenile VKH disease is very rare, yet when it occurs, ophthalmologic examinations help us diagnose and treat it.

## 1. Introduction

Vogt-Koyanagi-Harada (VKH) disease [1, 2] is one of the systemic autoimmune diseases. It is known that patients with VKH disease often suffer bilateral uveitis accompanied with serous retinal detachments and papillitis, following aseptic meningitis and deafness at early phase, and if the treatments are deficient or the induction of the treatments is delayed, they suffer poliosis and vitiligo at late phase. The onset age of VKH disease tends to be approximately from 20 to 50 years. Therefore, VKH disease in children is very rare [1]. We experienced an eight-year-old girl affirmatively diagnosed with VKH disease.

## 2. Case Report

**Figure 1:**
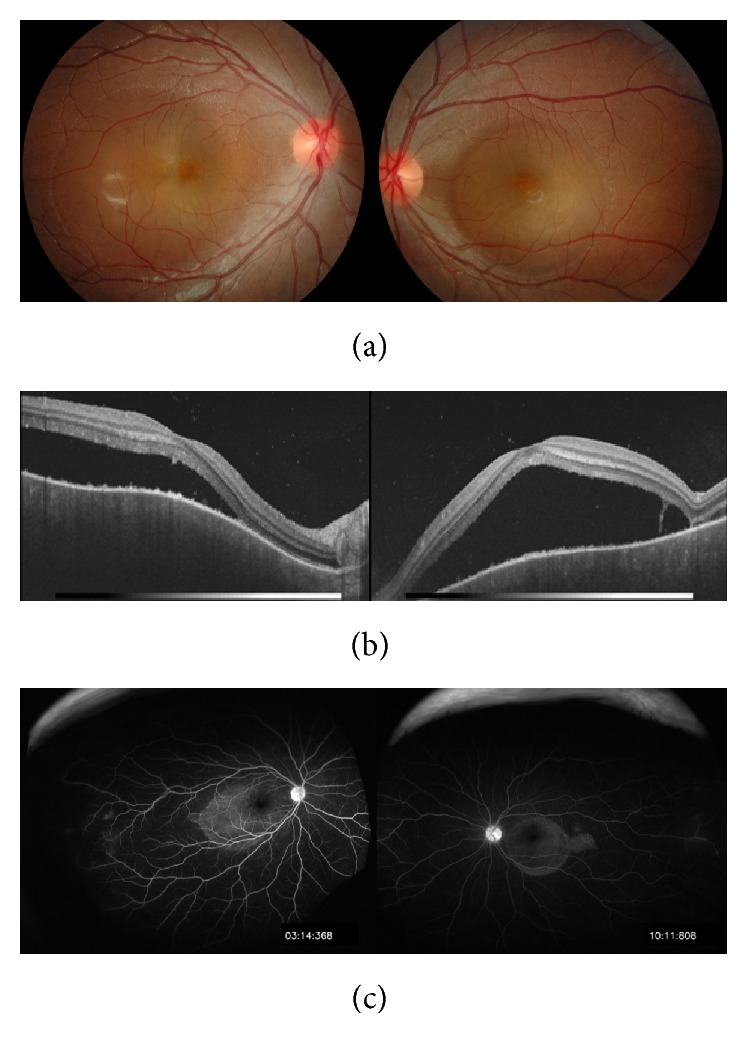
(a) Color photographs of the fundi at the first visit: serous retinal detachment of posterior pole and papillitis were observed in both eyes. (b) Ocular coherent tomography (OCT) images of the fundi at the first visit: OCT image revealed serous retinal detachment in both eyes. (c) Fluorescein angiography of the fundi at the first visit: fluorescein angiography revealed serous retinal detachment of posterior pole and papillitis in both eyes.

**Figure 2:**
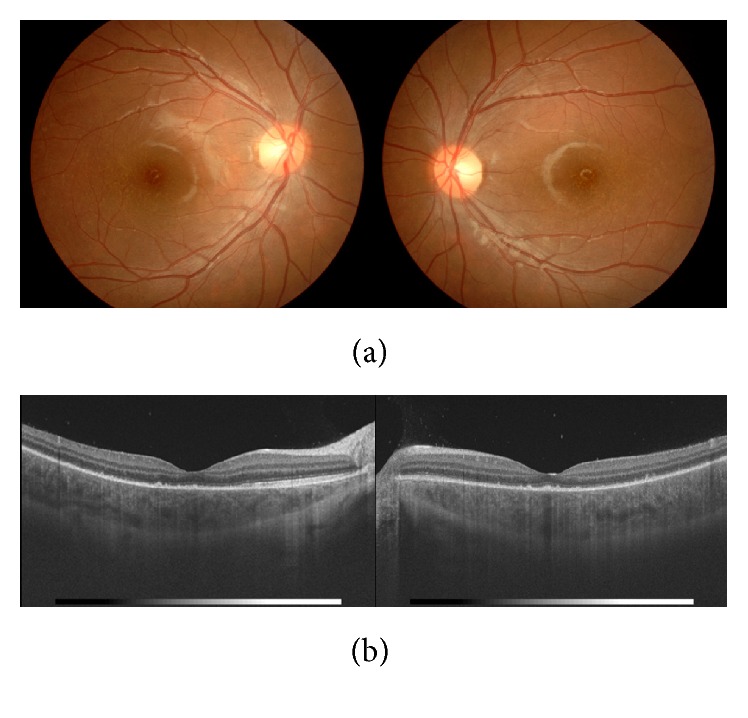
(a) Color photographs of the fundi at three weeks after the start of the treatments: in both eyes, serous retinal detachment of posterior pole and papillitis disappeared. (b) OCT images of the fundi at three weeks after the start of the treatments: in both eyes, serous retinal detachment of posterior pole disappeared and the thickness of choroid of posterior pole increased.

**Figure 3:**
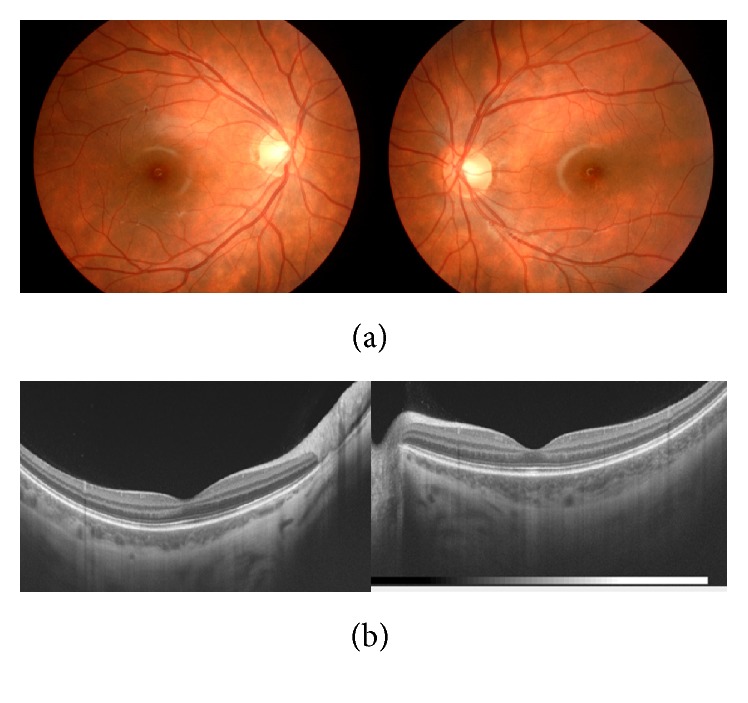
(a) Color photographs of the fundi at four months after the start of the treatments: both eyes showed sunset glow fundi. (b) OCT images of the fundi at four months after the start of the treatments: there was no serous retinal detachment of posterior pole and the thickness of choroid decreased in each eye.

An eight-year-old girl was referred to our hospital from a local ophthalmologist, claiming that she developed VKH. She had suffered reduced visual acuity of both eyes several days after influenza-like headache. She had an anamnesis of herpes labialis. At the first visit, her best-corrected visual acuity (BCVA) was 20/60 in the right eye and 20/1000 in the left eye, respectively. The intraocular pressures (IOPs) of the right and left eyes were 14 mmHg and 19 mmHg, respectively. Slight conjunctival hyperemia and mild inflammation (aqueous cell and flare) in the anterior chamber and vitreous body were observed in both eyes. Moreover, fundus examination (Figure 1), fluorescein angiography (FA) (Figure 2), and ocular coherent tomography (OCT) (Figure 3) revealed serous retinal detachment of posterior pole and papillitis in both eyes.

The body temperature was 36.8°C. Blood examinations revealed the following data: white blood cell 9.6 × 10^3^ cells/*μℓ*, CRP 0.02 mg/d*ℓ*, erythrocyte sedimentation rate 4 mm/hour, serous calcium 10.1 mg/d*ℓ*, angiotensin-converting enzyme 13.0 mU/m*ℓ*, and blood sugar 80 mg/d*ℓ*. Rheumatoid factor and antinuclear antibody were negative. Moreover, serous immunoglobulin M of herpes simplex, varicella zoster virus, and cytomegalovirus were all negative. Treponema pallidum hemagglutination test was negative. As for human lymphocyte antigens (HLA), she did not have HLA-DR4. Cerebrospinal fluid examination revealed that she suffered aseptic meningitis (76 cells/*μℓ*). Audiometry revealed that she had no deafness. Tuberculin test was not performed.

Judging from ocular symptoms and cerebrospinal fluid examination, she was diagnosed as suffering VKH disease. Therefore, according to the recommendation of the pediatrician, she was treated with corticosteroid pulse therapy (methylprednisolone 30 mg/kg/day, 3 days) followed by oral corticosteroid therapy (prednisolone 20 mg/day), which was tapered off and terminated seven months after. At three weeks after the start of the treatments, her decreased BCVA was restored to 20/20 in both eyes, while she had a recurrence of herpes labialis. In both eyes, serous retinal detachment of posterior pole and papillitis disappeared, and yet the thickness of choroid of posterior pole remained increased (Figures 2(a) and 2(b)). Four months after the start of the treatments, she had sunset glow fundi (Figure 3(a)), while OCT image showed that, in each eye, there was no serous retinal detachment of posterior pole and the thickness of choroid decreased (Figure 3(b)). Oral prednisolone was tapered slowly and terminated in seven months. The BCVA of the right and left eye enjoyed 20/20 and 20/20, respectively. For seven months after the induction of the treatments, she had no relapse of intraocular inflammation. Furthermore, she has not yet suffered from integumentary symptoms, such as alopecia, poliosis, and vitiligo, after the onset of ocular inflammation.

## 3. Discussion

According to VKH committee's revised diagnostic criteria [2], patients with complete VKH disease have neither history of ocular trauma (surgery) nor evidence suggestive of other ocular disease entities, and they have bilateral uveitis with early manifestations (diffuse choroiditis, subretinal fluid, and serous retinal detachments) or late manifestations (ocular depigmentation, chorioretinal depigmented scars, and recurrent or chronic anterior uveitis), neurological/auditory findings (aseptic meningitis and tinnitus), and integumentary symptoms (alopecia, poliosis, or vitiligo) after the onset of ocular inflammation [2]. The current patient revealed bilateral uveitis with early and late manifestations and aseptic meningitis devoid of dermal symptoms. Therefore, she was diagnosed with incomplete VKH disease, only lacking dermal symptoms.

The onset of VKH disease is usually in the second to fifth decade of life [1], thus pediatric VKH patients are very rare. To the best of our knowledge, the youngest patient is three-year-old boy reported by Takada et al. [3]. He was diagnosed with VKH disease by ocular symptoms (serous retinal detachments and papillitis and inflammation of anterior chamber), aseptic meningitis, and OCT images, although fluorescent fundus angiography was not performed.

Various clinical manifestations in pediatric VKH include headache and bilateral uveitis such as conjunctival hyperemia and serous retinal detachment [3–6], although manifest of a preceding fever is rare [6]. The current patient also revealed various clinical manifestations such as headache without a preceding fever, bilateral visual loss, and bilateral uveitis. And as for HLA, it was known that the prevalence of the HLA-DR4 antigen was 94% among all patients with VKH disease in Japan [1]. However, she did not have HLA-DR4, thus causing our decision making to be somewhat difficult. Moreover, it was reported that VKH disease in children, compared to adults, appeared to be more aggressive and severe [7–9]. In particular, FA and cerebrospinal fluid examination for children are difficult to perform. However, the definitive diagnosis of pediatric VKH disease is universally important for choosing aggressive modalities such as corticosteroid pulse therapy. Fortunately, in the early stage, she was diagnosed with VKH disease due to thorough examinations such as examination of cerebrospinal fluid, FA, and OCT. Consequently, timely induction of the proper therapy ensured good visual prognoses accompanied by no serious complications.

We have to bear in mind that juvenile VKH disease is very rare, yet it sometimes occurs. Therefore, careful and thorough examinations are nevertheless crucial in making the definitive diagnosis of VKH disease and good visual prognoses.
